# Human aldose reductase unfolds through an intermediate

**DOI:** 10.12688/f1000research.18963.2

**Published:** 2019-11-05

**Authors:** Gurprit Sekhon, Ranvir Singh

**Affiliations:** 1 Department cum National Center for Human Genome Studies and Research, Panjab University, Chandigarh, 160014, India

**Keywords:** Aldose reductase, Protein unfolding, Folding intermediate, Cooperativity, Tryptophan fluorescence, ANS fluorescence, Thermal denaturation

## Abstract

**Background:** Human aldose reductase (hAR) is the first and rate-limiting enzyme of the polyol pathway. For the development of secondary complications of diabetes in chronic hyperglycemic conditions, one of the critical factors is the increased flux of glucose through the polyol pathway.  Due to this clinical implication, hAR attracted considerable attention from the drug discovery perspective. In spite of extensive characterization in the context of biochemical and structural aspects, we know very little about the unfolding behavior of hAR. This study reports equilibrium unfolding studies of hAR.

**Methods:** We carried out thermal denaturation and chemical-induced equilibrium unfolding studies of hAR monitored by circular dichroism and fluorescence spectroscopy.

**Results:** Thermal denaturation studies presented a classical picture of two-state unfolding from native to the denatured state. The data was used to derive thermodynamic parameters and study the thermostability of hAR. Chemical induced equilibrium unfolding studies led us to discover an intermediate state, which gets populated at 3.5-4.0 M and 0.7-2.0 M of urea and GuHCl, respectively. Thermodynamic parameters derived from chemical-induced unfolding are in agreement with those obtained from thermal denaturation of hAR.

**Conclusion:** This study revealed that aldose reductase unfolds from native to the unfolded state via an intermediate. Assessment of the thermodynamic stability of native, intermediate, and unfolded states shows that significant energy barriers separate these states, which ensures the cooperativity of unfolding. As hAR functions in cells that are under osmotic and oxidative stress, these *in vitro* findings may have implications for its native conformation under the physiological state.

## Abbreviations

GuHCl, guanidine hydrochloride; TCEP, (tris(2-carboxyethyl)phosphine); ANS, 8-anilino-1-naphthalenesulfonic acid ammonium salt; IPTG, isopropyl β-D-1-thiogalactopyranoside; Trp, tryptophan.

## Introduction

Human aldose reductase (hAR) (EC 1.1.1.21) is an NADPH-dependent oxidoreductase that belongs to the superfamily of aldo-keto reductases ^1^. hAR is the first and rate-limiting enzyme of the polyol pathway and converts glucose to sorbitol ^2^. The polyol pathway is up-regulated under hyperglycemic conditions, and a significant proportion of glucose gets fluxed through this pathway, which leads to the accumulation of sorbitol, consumption of NADPH, and redox imbalance of NADPH/NADP ^+^ ratio. All these factors have been linked with various tissue-based pathologies associated with secondary complications of diabetes mellitus ^3^. Due to its clinical importance from the perspective of developing potent inhibitors to prevent or delay the onset of secondary diabetic complications, hAR has been widely studied in almost every aspect ^4^.

Extensive information is available in the literature about the structure and function of hAR, mainly related to active site of hAR from high-resolution crystal structures with a number of potential inhibitors ^5^, flexibility in the hAR binding site pocket ^6^ and the thermodynamics of closing/opening of the specificity pocket within binding site pocket of hAR ^7^. There is little investigation related to the folding/unfolding mechanism of hAR.

Protein folding is a fundamental process in all living systems. The linear sequence of amino acids encodes the information required for a polypeptide to fold. Despite extensive research and progress made over the years to understand this fundamental process, a complete understanding of protein folding mechanism(s) and the folding code remains elusive ^8^. Various models have been proposed to explain the mechanism underlying protein folding reaction from time to time ^9^. ‘New view folding model’ based on energy landscape theory (ELT) and ‘defined pathways model’ based on the concept of foldons are current alternatives to explain the protein folding mechanism. ^10^.

Among several hallmarks of protein folding are its spontaneity and cooperativity, along with fast reaction timescale. Usually, a small free energy change is required to shift the equilibrium between the folded and unfolded state ensemble. Complex cellular solvation environment plays a vital role in regulating the equilibrium between folded and unfolded ensemble in vivo, which are separated by small free energy barrier (~5 - 10 Kcal mol-1) ^11^.

Under physiological conditions, protein structure fluctuates among different native conformations separated by close free energy barriers ^12^. Since hAR activity leads to sorbitol accumulation, leading to osmotic stress, it seems to function under stress conditions which might perturb its native conformation ensemble. Here we report on thermal denaturation and chemical induced unfolding studies of hAR. Thermal denaturation revealed a simple two-state transition, whereas chemical induced unfolding led us to discover an intermediate state during hAR unfolding.

## Methods

### Materials

All chemicals were reagent grade and purchased from Sigma-Aldrich.

### Protein purification

The hAR cDNA cloned into expression vector pET-15b (Novagen) was a kind gift from Dr. Alberto Podjarny (Department of Integrated Structural Biology, Institut de Génétique et de Biologie Moléculaire et Cellulaire, CNRS, INSERM, France). The plasmid, coding for a hexahistidine-tagged protein, was expressed into *E. coli* strain BL21 (DE3) (Novagen). The cells with recombinant plasmid were grown with 100 μM ampicillin at 37°C to an OD _600_ nm value of 0.7, and protein expression was induced by adding 1 mM IPTG. Cells were grown for a further 3 hours at 37°C. All further operations were carried out at 4°C unless otherwise stated. Cells were centrifuged, resuspended, and lysed by sonication. A Ni-NTA affinity column (GE Healthcare) was used for protein purification. The material used for the stationary phase for the column was Ni-Sepharose, and the flow rate of the column was adjusted to 0.5 ml min ^-1^. Imidazole and other salts were removed by repeated dialysis in 50 mM potassium phosphate, pH-7 buffer containing 50 mM NaCl. Protein concentration was estimated using the molar extinction coefficient and absorbance reading at 280 nm. The histidine tag from recombinant protein was removed by thrombin (4 units of thrombin per mg of recombinant protein at room temperature for 3 hours). The cleaved protein was passed through the Ni-NTA column, and purified protein without tag was collected as flow-through. Enzyme activity was checked as per standard assay ^13^. We analyzed the homogeneity and molecular weight of hAR with and without histidine tags under denaturing conditions on 15% SDS-PAGE. Purified hAR was stored at -20°C for further studies.

### Thermal denaturation

Thermal denaturation was carried out at a final concentration of 2.8 µM protein in 50 mM potassium phosphate buffer, pH 7.0, containing 50 mM NaCl and 0.1mM TCEP. The transition between 20–70 °C was followed using a far-UV circular dichroism (CD) signal at 222 nm by using a 0.1 cm path-length cuvette at a sampling rate of 1.0 °C min ^-1^ in a Jasco J-810 spectropolarimeter. After subtracting buffer blank, we have reported the change in ellipticity (millidegree) at 222 nm.

### Chemical induced unfolding

To follow chemical induced unfolding, we prepared samples with 1.4 μM protein concentration in phosphate buffer (described earlier), containing different concentrations of GuHCl/urea. Samples were incubated for 12 hours to reach equilibrium at 25°C, after which no change in signal occurred either in fluorescence or CD spectra. Trp fluorescence (excitation at 295 nm and emission recorded between 300 nm to 400 nm) and ANS fluorescence (excitation at 370 nm and emission recorded between 400 nm to 600 nm) measurements were performed using a Hitachi F-7000 fluorescence spectrophotometer for GuHCl samples and Jasco J-815 spectropolarimeter for urea samples. Far-UV CD measurements were performed using Jasco J-810 spectropolarimeter for GuHCl samples and Jasco J-815 spectropolarimeter for urea samples. Quartz cuvette of 1 cm and 0.5 cm path-length were used for fluorescence and CD measurements, respectively. All measurements were done at 25 °C. We have reported spectra as ellipticity (millidegree) after baseline correction.

### Heat capacity change (Δ *C _p_*) calculations

We have calculated the value of Δ *C _p_* for the unfolding of hAR from the change in accessible surface area (ΔASA) according to Equation 1 ^14^

$\Delta C_{p}=-251+0.19\times [\Delta \text{ASA}]\;(Equation 1)$

ProtSA webserver was used to calculate the change in accessible surface area from native to the unfolded conformation of hAR ^15^.

### Data analysis

GraphPad Prism version 7.04 for Windows (GraphPad Software, La Jolla, California) was used for the analysis of thermal denaturation and chemical-induced unfolding data based on two-state and three-state models respectively, as described in following sections.

### Thermal denaturation

The Least-square analysis was used to fit the data to Equation 2 ^16^.

$Y=\frac{(A_{n}+b_{N}\times T)+(A_{u}+b_{U}\times T)\times exp\left(\left(\frac{\Delta H_{g}}{R}\right)\times \left(\frac{1}{T_{g}}-\frac{1}{T}\right)\right)}{1+\exp\left(\left(\frac{\Delta H_{g}}{R}\right)\times \left(\frac{1}{T_{g}}-\frac{1}{T}\right)\right)}\;(Equation 2)$

Where *A _n_* and *A _u_* are native and unfolded state baseline intercepts, respectively, and *b _N_* and *b _U_* are native and unfolded baseline slopes, respectively. Δ *H _m_* is enthalpy change at melting temperature ( *T _g_*). *T* is absolute temperature, and R is the gas constant.

### Calculation of *ΔG* values for the transition region

Signal for native ( *Y _N_ = A _n_ + b _N_* × *T*) and unfolded baseline ( *Y _U_ = A _u_ + b _U_* × *T*) for every point in transition region was calculated from Equation 2. If *Y* is signal for a particular point in transition region, then the fraction of unfolded protein ( *F _u_*) at this point is given by Equation 3.

$F_{u}=\frac{Y_{n}-Y}{Y_{n}-Y_{u}}\;(Equation 3)$

The equilibrium constant (k) was calculated from the relative population of species using Equation 4.

$k=\frac{F_{u}}{1-F_{u}}\;(Equation 4)$

Δ *G* was calculated as a function of temperature using Equation 5.

ΔG=−RTlnk(Equation 5)

### Thermal stability curve

The thermal stability curve of hAR was constructed based on Equation 6–8 ^17^.

$\Delta H_{T}=\Delta H_{g}+\Delta C_{p}(T-T_{g})\;(Equation 6)$

$\Delta S_{T}=\frac{\Delta H_{g}}{T_{g}}+\Delta C_{p}\times LN\left(\frac{T}{T_{g}}\right)\;(Equation 7)$

$\Delta G_{s}=\Delta H_{g}\times \left(1-\frac{T}{T_{g}}\right)+\Delta C_{p}\times \left(T-T_{g}-\left(T\times LN\left(\frac{T}{T_{g}}\right)\right)\right)\;(Equation 8)$

Where Δ *H _T_* and Δ *S _T_* are enthalpy change and entropy change, respectively, at temperature *T* with reference to *T _g_*. *T _h_*, *T _s_* and *T _g_* are the temperatures at which Δ *H*, Δ *S*, and Δ *G* are zero, respectively. Δ *G _s_* is the stabilization free energy of the native state relative to the unfolded state.

### Chemical induced unfolding

The least-square analysis was used to fit the data to Equation 9 ^16^.

$Y=\frac{(A_{n}+b_{N}\times [D])+(A_{i}+b_{I}\times [D])\times \exp\left(-\left(\frac{\Delta G_{\left(N-I\right)}}{R*T}-\frac{m_{{\left(N-I\right)}^{\times [D]}}}{R*T}\right)\right)+(A_{u}+b_{U}\times [D])\times \exp\left(-\left(\frac{\Delta G_{\left(N-I\right)}}{R\times T}-\frac{m_{{\left(N-I\right)}^{*\;[D]}}}{R\times T}\right)\right)\times \exp\left(-\left(\frac{\Delta G_{\left(I-U\right)}}{R\times T}-\frac{m_{{\left(I-U\right)}^{\times [D]}}}{R\times T}\right)\right)}{1+\exp\left(-\frac{\Delta G_{\left(N-I\right)}}{R\times T}-\frac{m_{{\left(N-I\right)}^{\times [D]}}}{R\times T}\right)-\exp\left(-\left(\frac{\Delta G_{\left(N-I\right)}}{R\times T}-\frac{m_{{\left(N-I\right)}^{\times [D]}}}{R\times T}\right)\right)\times \exp\left(-\left(\frac{\Delta G_{\left(I-U\right)}}{R\times T}-\frac{m_{{\left(I-U\right)}^{\times [D]}}}{R\times T}\right)\right)}\;(Equation 9)$

Where *A _n_*, *A _u_*, and *A _i_* are the native, unfolded, and intermediate baseline intercepts, respectively, and b _N_, *b _U_*, and *b _I_* are the native, unfolded, and intermediate baseline slopes, respectively. [ *D*] is denaturant concentration in a molar unit. *m _(N-I)_* and *m _(I-U)_* are denaturant gradient for native to intermediate and intermediate to unfolded state, respectively. Δ *G _(N-I)_* and Δ *G _(I-U)_* are stabilization free energy of intermediate state relative to the native and unfolded state, respectively.

## Results

### Thermal denaturation monitored by far-UV CD

**Figure 1.**
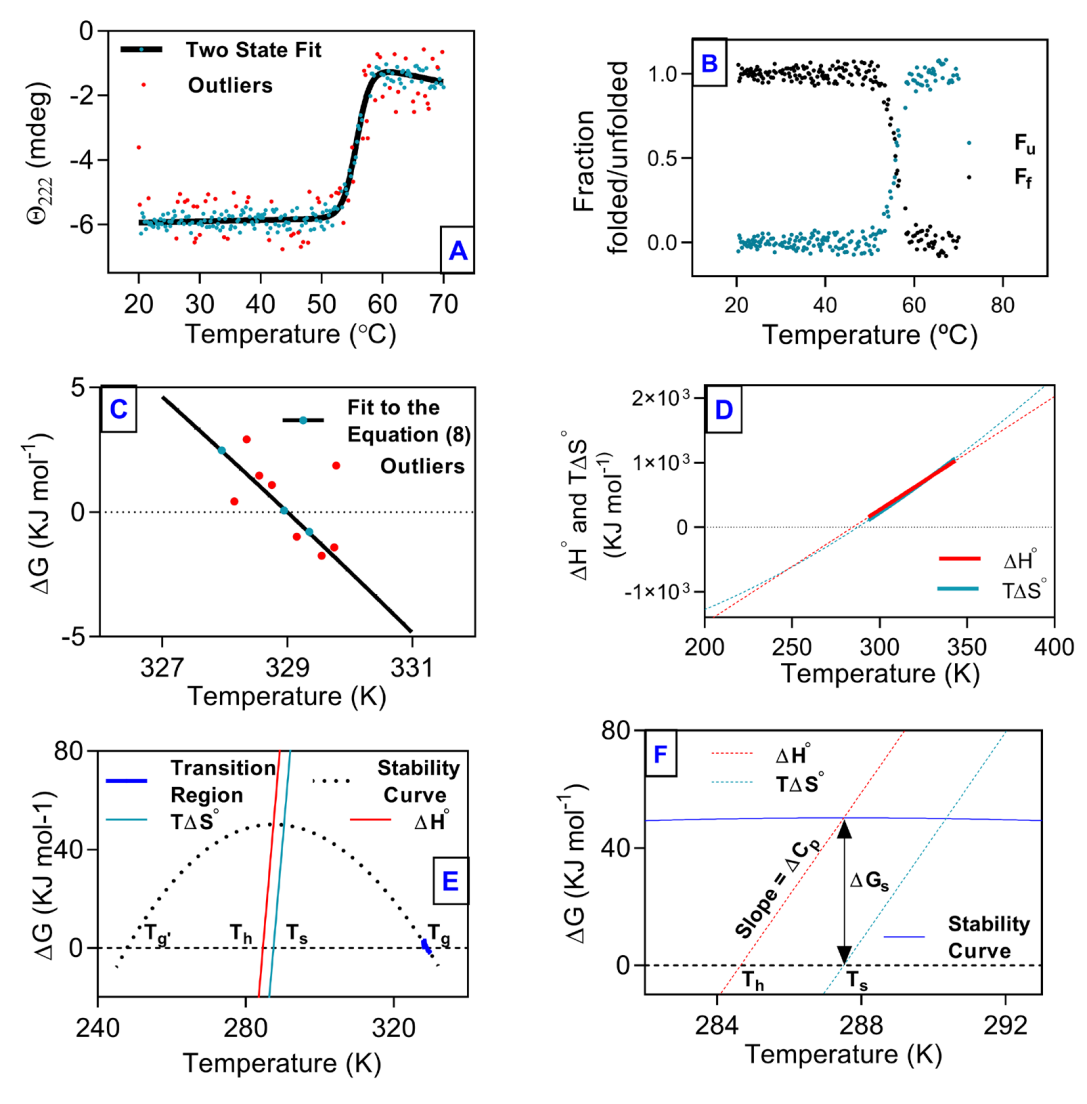
Thermal denaturation studies of human aldose reductase (hAR) monitored by far-UV circular dichroism. ( **A**) Change in ellipticity at 222 nm plotted as a function of temperature. ( **B**) The fraction of protein folded (black dots) and unfolded (cyan dots) plotted against temperature. ( **C**) The portion of the transition curve used in van't Hoff analysis. ( **D**) Plots of ΔH and ΔS as a function of temperature. ( **E**) Thermal stability curve of hAR. ( **F**) Triangular relationship among T _h_, T _s_, and ∆G _s_. The explanation for T _g_, T _g’_, T _h_, and T _s_ is given in the text. Dashed lines are extrapolations.The solid lines represent curves fitted to the unfolding transition; filled symbols represent data points from unfolding experiments; and red symbols represent outliers.

Change in ellipticity at 222 nm fitted well based on a two-state model ( Figure 1A). This analysis gave values for Δ *H _g_* and *T _g_*, which along with the Δ *C _p_* value calculated from Equation 1 were used for non-linear regression of the transition region (±5 kJ mol ^-1^) to Equation 8 ( Figure 1C). Values of Δ *H* and Δ *S* were calculated over an extended range of temperatures by using Equation 7 and Equation 8, respectively ( Figure 1D). The thermal stability curve is an extrapolation of transition region, assuming constant Δ *C _p_* during the unfolding transition ( Figure 1E). The relationship between *T _s_*, *T _h_* and Δ *G (T _s_ – T _h_ =* Δ *G _s_*/Δ *C _p_)* is presented in Figure 1F. Thermodynamic parameters obtained from the analysis of thermal denaturation data are listed in Table 1. All raw data are available as *Underlying data* ^18^.

**Table 1.** Thermodynamic parameters derived from equilibrium unfolding transitions of hAR during thermal denaturation and Chemical induced unfolding studies using different probes.

Chemical unfolding	Probe	ΔG _(N-I)_ (KJ mol ^-1^)	ΔG _(I-U)_ (KJ mol ^-1^)	m _(N-I)_ (KJ mol ^-1^ M ^-1^)	m _(I-U)_ (KJ mol ^-1^ M ^-1^)	C _m(N-I)_ (M)	C _m(I-U)_ (M)		
	GuHCl [F _314_]	9.47 ± 1.69	58.34 ± 1.14	48.34 ± 7.69	23.56 ± 0.46	0.16	2.48		
	GuHCl-ANS [F _480_]	16.48 ± 0.54	57.26 ± 3.33	38.99 ± 1.15	22.38 ± 1.29	0.42	2.56		
	Urea [F _314_]	30.88 ± 4.47	35.04 ± 4.16	13.11 ± 2.01	7.99 ± 0.84	2.35	4.49		
	Urea-ANS [F _480_]	28.61 ± 2.39	38.6 ± 2.14	12.1 ± 1.13	8.95 ± 0.46	2.36	4.32		
	GuHCl [Θ _219_]	13.68 ± 5.78	34.13 ± 3.75	32.17 ± 11.63	14.9 ± 1.58	0.43	2.29		
	Urea [Θ _221.8_]	30.53 ± 6.16	24.43 ± 6.25	14.12 ± 3.03	5.99 ± 1.30	2.16	4.08		
Thermal denaturation	Probe	ΔG _u_ (KJ mol ^-1^)	T _g_ (K)	ΔC _p_ (KJ mol ^-1^ K ^-1^)	ΔH _g_ (KJ mol ^-1^)	ΔS _m_ (KJ mol ^-1^)	T _g`_ (K)	T _h_ (K)	T _s_ (K)
	Temperature [Θ _222_]	50.25	329 ± 0.01	17.57	779.20 ± 15.36	2.37	248	284.65	287.51

### Chemical induced unfolding monitored by fluorescence

There are six Trp residues in hAR, out of which four are part of the hydrophobic active site pocket in the core of the β-barrel, and two are buried in alpha-helices surrounding the barrel. Their fluorescence provided a global signal of change in the tertiary structure. ANS has been extensively used as a probe for non-native, partially unfolded conformations of the protein. The binding of ANS to hydrophobic regions results in a significant enhancement of ANS fluorescence and a pronounced blue-shift of the *λ*_max_^19^.

**Figure 2.**
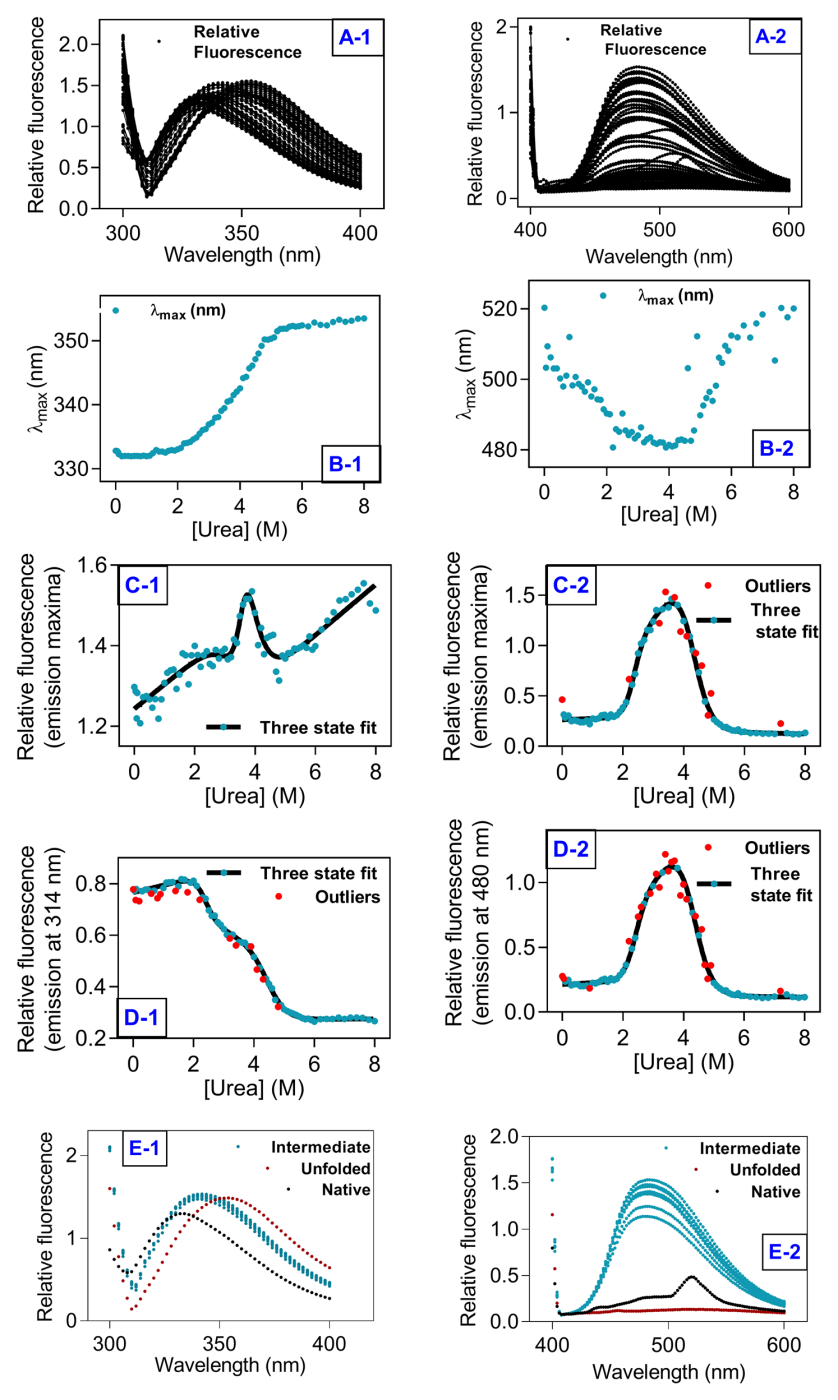
Urea induced unfolding studies of human aldose reductase (hAR)monitored by fluorescence. ( **A1**) Trp fluorescence scans and ( **A2**) ANS fluorescence scans for all the samples. ( **B1**) λ _max_ (Trp fluorescence) and ( **B2**) λ _max_ (ANS fluorescence) against [urea]. ( **C1**) I _max_ (Trp fluorescence) and ( **C2**) I _max_ (ANS fluorescence) against [urea]. ( **D1**) I _295/314_ (Trp fluorescence) and ( **D2**) I _370/480_ (ANS fluorescence) against [urea]. ( **E1**) Trp fluorescence and ( **E2**) ANS fluorescence of samples in native (black), intermediate (cyan), and unfolded state (red). Solid lines represent curves fitted to the unfolding transitions; filled symbols represent data points from unfolding experiments; red symbols represent outliers.

**Figure 3.**
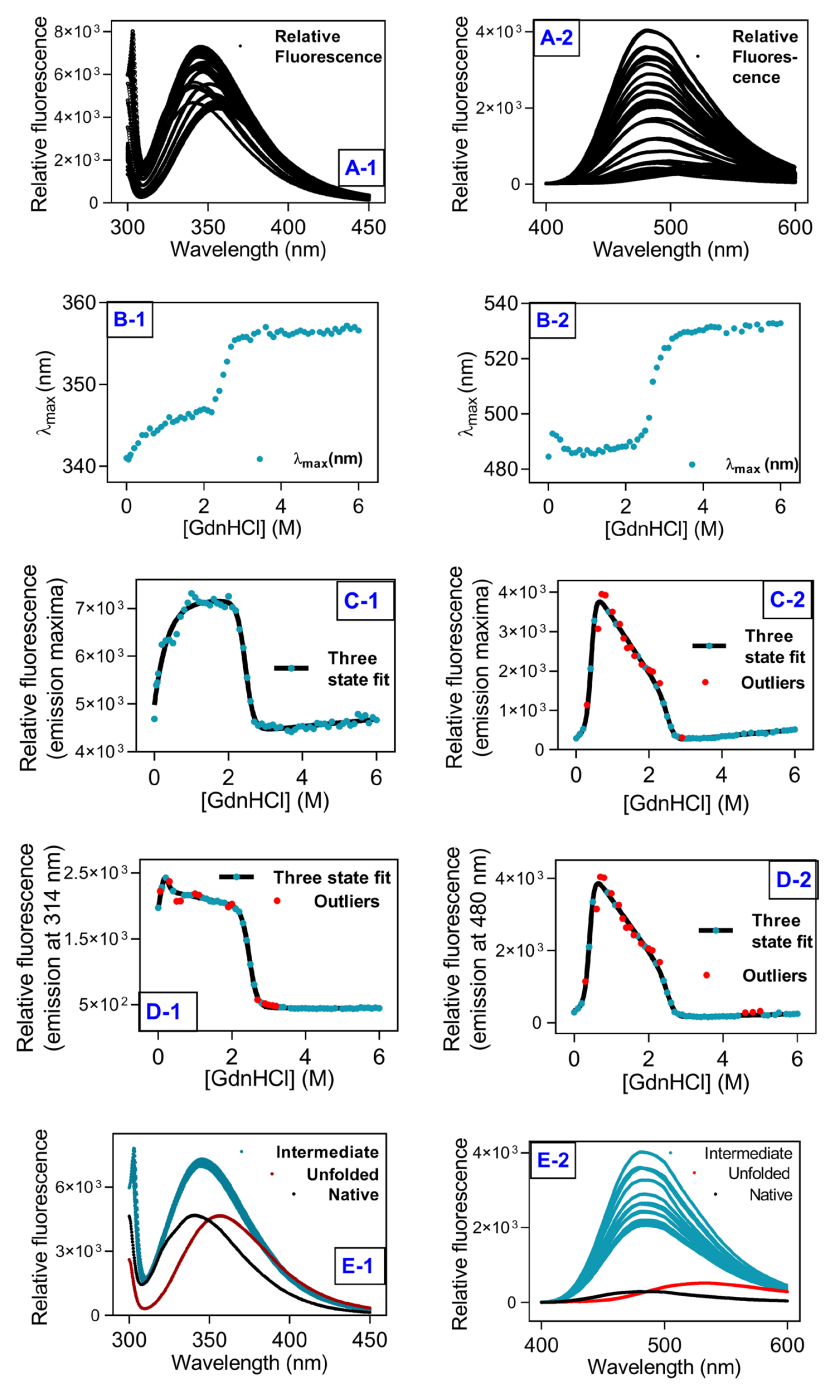
GuHCl-induced unfolding studies of human aldose reductase (hAR)monitored by fluorescence. ( **A1**) Trp fluorescence scans and ( **A2**) ANS fluorescence scans. ( **B1**) λ _max_ (Trp fluorescence) and ( **B2**) λ _max_ (ANS fluorescence) against [GuHCl]. ( **C1**) I _max_ (Trp fluorescence) and ( **C2**) I _max_ (ANS fluorescence) against [GuHCl]. ( **D1**) I _295/314_ (Trp fluorescence) and ( **D2**) I _370/480_ (ANS fluorescence) against [GuHCl]. ( **E1**) Trp fluorescence and ( **E2**) ANS fluorescence of samples in native (black), intermediate (cyan), and unfolded state (red). Solid lines represent curves fitted to the unfolding transitions; filled symbols represent data points from unfolding experiments; red symbols represent outliers.

Fluorescence emission profiles of hAR equilibrated with different concentrations of denaturants are presented in Figure 2 ( Figure 2A and Figure 3A for urea and GuHCl, respectively). A plot of *λ*_max_ against denaturant concentration indicated a cooperative transition from native to unfolded state ( Figure 2B and Figure 3B for urea and GuHCl, respectively). In the case of ANS fluorescence, a significant blue-shift of around 20 nm and 10 nm from the native to the intermediate state was observed for urea and GuHCl, respectively ( Figure 2B2 and Figure 3B2 for urea and GuHCl, respectively).

A plot of *I*_max_ against denaturant concentration indicated the presence of an intermediate during unfolding transition ( Figure 2C and Figure 3C for urea and GuHCl, respectively). *I*_max_ in case of ANS fluorescence fits satisfactorily based on the three-state model ( Figure 2C2 and Figure 3C2 for urea and GuHCl, respectively). For both urea and GuHCl induced unfolding, Trp fluorescence emission intensity at 314 nm fits satisfactorily to the three-state model. In the case of ANS fluorescence, both *I _max_* and fluorescence emission intensity at 480 nm fit equally well based on three-state model. Thus, Trp fluorescence emission intensity at 314 nm and ANS fluorescence emission intensity at 480 nm were analyzed based on the three-state model to evaluate the thermodynamic stability of hAR ( Figure 2D and Figure 3D for urea and GuHCl, respectively). The thermodynamic parameters obtained from the fittings are listed in Table 1. Trp and ANS fluorescence demonstrate the presence of an intermediate state populated at 3.5-4.0 M and 0.7-2 M urea and GuHCl concentration, respectively, apart from the native and unfolded states ( Figure 2E and Figure 3E for urea and GuHCl, respectively).

### Chemical induced unfolding monitored by far-UV CD

**Figure 4.**
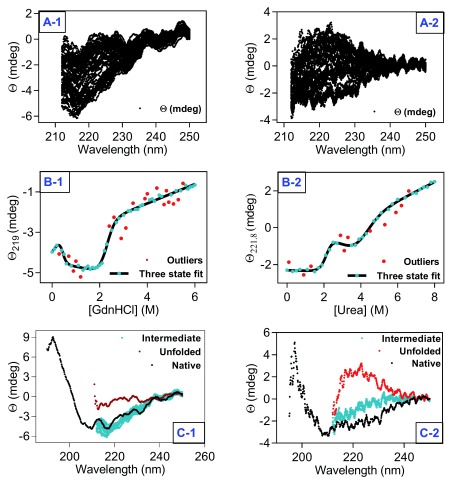
GuHCl/urea induced unfolding studies of human aldose reductase (hAR)monitored by far-UV circular dichroism (CD). ( **A1**) far-UV CD scans recorded for GuHCl and ( **A2**) far-UV CD scans recorded for urea for all the samples. ( **B1**) change in ellipticity at 219 nm against [GuHCl] and ( **B2**) change in ellipticity at 221.8 nm against [urea]. ( **C**) CD spectra of samples in native (black), intermediate (cyan), and unfolded state (red) for ( **C1**) GuHCl and ( **C2**) urea, respectively. The solid lines represent curves fitted to the unfolding transitions; filled symbols represent data points from unfolding experiments; red symbols represent outliers in data fitting.

Unfolding profiles of hAR equilibrated at different denaturant concentrations monitored by far-UV CD are presented in Figure 4A1 and 4A2 for GuHCl and urea, respectively. The thermodynamic stability of hAR was determined based on the three-state model by plotting the change in ellipticity at 219/222 nm as a function of denaturant concentration ( Figure 4B1 and 4B2 for GuHCl and urea, respectively). The transition determined by far-UV CD detected intermediate state at similar concentrations of denaturant as interrogated by fluorescence spectroscopy. Far-UV CD profiles clearly distinguish three states ( Figure 4C1 and 4C2 for GuHCl and urea, respectively). Thermodynamic parameters derived from far-UV CD data are listed in Table 1.

## Discussion

The intermediate state with enhanced ANS fluorescence and significant blue shift of λ _max_ pointed to an intermediate state with ‘molten like’ nature during the chemical-induced unfolding of hAR. Far-UV CD studies strongly suggest that the intermediate state retains significant secondary structure during urea- and GuHCl-induced unfolding.

During chemical induced unfolding, hAR unfolds through an intermediate state, which is absent during thermal denaturation. Moderate concentration of denaturant is known to stabilize native or intermediate state ^20^. The absence of such a stabilizing agent may be the reason that we could not detect the intermediate state during thermal denaturation.

In all three probes used in studying unfolding, the value of Δ *G*_( *N*- *I*)_ obtained is ~30 kJ mol ^-1^ and ~15 kJ mol ^-1^ for urea- and GuHCl-induced unfolding, respectively while a Δ *G _s_* of ~70 kJ mol ^-1^ is almost same for both denaturants ( Table 1). Thus, while urea seems to stabilize the native state with respect to the intermediate state, GuHCl seems to stabilize the intermediate state with respect to the native state.

Small molecules change the free energy landscape of protein upon binding by selectively stabilizing native or intermediate/unfolded state ^21^. The difference in the value of Δ *G _s_* obtained from thermal denaturation and chemical-induced unfolding is ~20 kJ mol ^-1^ ( Table 1), which is most likely due to free energy of stabilization and destabilization by urea and GuHCl, respectively.

Values of Δ *G _(N-I)_* obtained from the analysis of ANS fluorescence data are 16.48 and 28.61 kJ mol ^-1^ for GuHCl and urea, respectively ( Table 1), which indicates that a steep energy barrier does not separate intermediate state from the native state. Values for Δ *G _(I-U)_* obtained from ANS fluorescence are 57.26 and 38.6 kJ mol ^-1^ for GuHCl and urea, respectively ( Table 1), which indicates that the intermediate state is separated from unfolded state by a high energy barrier. Thus, the intermediate state of hAR is close to its native state, which makes it functionally more relevant. These studies are essential to access the effect of other physiological relevant molecules on hAR stability ^22^. Protein stability of hAR in the cellular environment under hyperglycemia-induced cellular stress conditions has to be studied to aid rational drug design ^23^.

In summary, equilibrium unfolding studies of hAR have led us to discover that hAR unfolds through an intermediate state, which is close to the native state, and might have physiological relevance under hyperglycemic conditions in diabetes.

## Data availability

### Underlying data

Figshare: data_f1000_hAR_unfolding.zip. https://doi.org/10.6084/m9.figshare.8001998.v1 ^18^.

This project contains raw data for thermal denaturation and chemical induced unfolding studies on human aldose reductase.

Data are available under the terms of the Creative Commons Attribution 4.0 International license (CC-BY 4.0).

## References

[1] Penning TM: The aldo-keto reductases (AKRs): Overview. *Chem Biol Interact.* 2015;234:236–246. 10.1016/j.cbi.2014.09.024 25304492 PMC4388799

[2] Brownlee M: The pathobiology of diabetic complications: a unifying mechanism. *Diabetes.* 2005;54(6):1615–1625. 10.2337/diabetes.54.6.1615 15919781

[3] Yan LJ: Redox imbalance stress in diabetes mellitus: Role of the polyol pathway. *Animal Model Exp Med.* 2018;1(1):7–13. 10.1002/ame2.12001 29863179 PMC5975374

[4] Maccari R Ottanà R: Targeting aldose reductase for the treatment of diabetes complications and inflammatory diseases: new insights and future directions. *J Med Chem.* 2015;58(5):2047–2067. 10.1021/jm500907a 25375908

[5] Howard EI Sanishvili R Cachau RE: Ultrahigh resolution drug design I: details of interactions in human aldose reductase-inhibitor complex at 0.66 A. *Proteins.* 2004;55(4):792–804. 10.1002/prot.20015 15146478

[6] Sotriffer CA Krämer O Klebe G: Probing flexibility and “induced-fit” phenomena in aldose reductase by comparative crystal structure analysis and molecular dynamics simulations. *Proteins.* 2004;56(1):52–66. 10.1002/prot.20021 15162486

[7] Rechlin C Scheer F Terwesten F: Price for Opening the Transient Specificity Pocket in Human Aldose Reductase upon Ligand Binding: Structural, Thermodynamic, Kinetic, and Computational Analysis. *ACS Chem Biol.* 2017;12(5):1397–1415. 10.1021/acschembio.7b00062 28287700

[8] Dill KA MacCallum JL: The protein-folding problem, 50 years on. *Science.* 2012;338(6110):1042–6. 10.1126/science.1219021 23180855

[9] Judy E Kishore N: A look back at the molten globule state of proteins: thermodynamic aspects. *Biophys Rev.* 2019;11(3):365–375. 10.1007/s12551-019-00527-0 31055760 PMC6557940

[10] Englander S Mayne L: The case for defined protein folding pathways. *Proc Natl Acad Sci USA.* 2017;114(31):8253–8258. 10.1073/pnas.1706196114 28630329 PMC5547639

[11] Gruebele M Dave K Sukenik S: Globular Protein Folding *In Vitro* and *In Vivo*. *Annu Rev Biophys.* 2016;45(1):233–251. 10.1146/annurev-biophys-062215-011236 27391927

[12] Cremades N Sancho J Freire E: The native-state ensemble of proteins provides clues for folding, misfolding and function. *Trends Biochem Sci.* 2006;31(9):494–496. 10.1016/j.tibs.2006.07.001 16870449

[13] Balendiran GK Sawaya MR Schwarz FP: The role of Cys-298 in aldose reductase function. *J Biol Chem.* 2011;286(8):6336–6344. 10.1074/jbc.M110.154195 21084309 PMC3057803

[14] Myers JK Pac CN Scholtz JM: Denaturant *m* values and heat capacity changes: relation to changes in accessible surface areas of protein unfolding. *Protein Sci.* 1995;4(10):2138–2148. 10.1002/pro.5560041020 8535251 PMC2142997

[15] Estrada J Bernadó P Blackledge M: ProtSA: a web application for calculating sequence specific protein solvent accessibilities in the unfolded ensemble. *BMC Bioinformatics.* 2009;10:104. 10.1186/1471-2105-10-104 19356231 PMC2674053

[16] Grimsley GR Trevino SR Thurlkill RL: Determining the conformational stability of a protein from urea and thermal unfolding curves. *Curr Protoc Protein Sci.* 2013;Chapter 28:Unit28.4. 10.1002/0471140864.ps2804s71 23377851

[17] Becktel WJ Schellman JA: Protein stability curves. *Biopolymers.* 1987;26(11):1859–1877. 10.1002/bip.360261104 3689874

[18] Sekhon G Singh R: data_f1000_hAR_unfolding.zip. *figshare.*Dataset.2019 10.6084/m9.figshare.8001998.v1

[19] Cattoni DI Kaufman SB González Flecha FL: Kinetics and thermodynamics of the interaction of 1-anilino-naphthalene-8-sulfonate with proteins. *Biochim Biophys Acta.* 2009;1794(11):1700–1708. 10.1016/j.bbapap.2009.08.007 19683079

[20] Bhuyan AK: Protein stabilization by urea and guanidine hydrochloride. *Biochemistry.* 2002;41(45):13386–13394. 10.1021/bi020371n 12416983

[21] Street TO Bolen DW Rose GD: A molecular mechanism for osmolyte-induced protein stability. *Proc Natl Acad Sci U S A.* 2006;103(38):13997–4002. 10.1073/pnas.0606236103 16968772 PMC1564065

[22] Kabir A Honda RP Kamatari YO: Effects of ligand binding on the stability of aldo-keto reductases: Implications for stabilizer or destabilizer chaperones. *Protein Sci.* 2016;25(12):2132–2141. 10.1002/pro.3036 27595938 PMC5119574

[23] Brylski O Ebbinghaus S Mueller JW: Melting Down Protein Stability: PAPS Synthase 2 in Patients and in a Cellular Environment. *Front Mol Biosci.* 2019;6:31. 10.3389/fmolb.2019.00031 31131283 PMC6509946

