# Supplementary material for: Human aldose reductase unfolds through an intermediate
Source: F1000Res. 2019 Nov 5;8:564. Originally published 2019 Apr 26. [Version 2] doi: 10.12688/f1000research.18963.2 (PMC6844136; doi:10.12688/f1000research.18963.2)
Supplement: Peer review history

# Peer review: Reviewer response for version 2

**DOI:** 10.5256/f1000research.23232.r56209

**Published:** 2019-11-14

**Authors:** Yong-Bin Yan

**Affiliations:** 1 State Key Laboratory of Membrane Biology, School of Life Sciences, Tsinghua University, Beijing, China

I am sorry for that I can not approve the revised versioon since the authors did not address most of my concerns raised during last round of review, particular of the change in enzymatic activity, the validity of the calculations and the effect of pathological stresses.

I confirm that I have read this submission and believe that I have an appropriate level of expertise to confirm that it is of an acceptable scientific standard, however I have significant reservations, as outlined above.

# Peer review: Reviewer response for version 2

**DOI:** 10.5256/f1000research.23232.r56208

**Published:** 2019-11-06

**Authors:** Jonathan W. Mueller

**Affiliations:** 1 Institute of Metabolism and Systems Research (IMSR), University of Birmingham, Birmingham, UK

The authors seem to have carefully addressed all points raised.

I confirm that I have read this submission and believe that I have an appropriate level of expertise to confirm that it is of an acceptable scientific standard.

# Peer review: Reviewer response for version 1

**DOI:** 10.5256/f1000research.20788.r51586

**Published:** 2019-08-01

**Authors:** Yong-Bin Yan

**Affiliations:** 1 State Key Laboratory of Membrane Biology, School of Life Sciences, Tsinghua University, Beijing, China

In this research, the authors studied the equilibrium unfolding pathway of human aldose reductase (hAR) by spectroscopic methods. Although this pure unfolding study may provide information of how hAR unfolds, the underlying physiological relevance is unclear. Furthermore, the present study needs to be improved in the quality of the data and interpretation.

     During my preparation of the comments, Professor Uversky and Professor Mueller have published their excellent comments and suggestions. I agree with those points raised in their reports. Below are some additional comments that could be considered by the authors to improve their work.

1. Although the authors have mentioned the importance of hAR in the Abstract and Introduction, especially for that it is an enzyme functions under osmotic stresses, it is unclear for the physiological relevance of the information obtained here using heat and chemical denaturants. I suggest the authors considered the physiologically relevant factors such as osmotic stress in their revised or future work.
2. hAR is an enzyme. I suggest the authors monitor the activity of the enzyme during unfolding to provide information regarding the local structural changes around the active site.
3. I am not convinced by the calculations of the thermodynamic parameters. The equations listed in the manuscript are derived from reverse folding processes. However, the authors did not show that the folding of hAR was reversible or not.
4. The authors concluded that the thermal unfolding of hAR was a two-state process by far-UV CD spectra analysis. However, only one method/probe is difficult for the definition of a two-state transition. It’s better to study the transition with two or more probes and pure two-state transition will give similar transitions as well as thermodynamic parameters for all probes.
5. The authors concluded that hAR undergo a three-state transition during unfolding by chemical denaturants. The present data could not support this statement. The emission maximum wavelength of the Trp fluorescence revealed no populations of any intermediates. An intermediate seems to populate at low denaturant concentrations when observed by Trp fluorescence intensity. However, intrinsic fluorescence intensity is easily affected by numerous environmental factors and is not as reliable as the emission maximum wavelength. Yes, there is an obvious increase in ANS fluorescence. Previously Turoverov and his co-authors have shown that the appearance of off-pathway aggregates, in some cases, can enhance ANS fluorescence. ^1^ It is necessary to avoid the misleading interpretation by identifying the potential intermediate(s) via more methods.
6. It is unclear whether all the solid lines are fitted data or not. Moreover, it is better to provide the residuals to facilitate the readers to evaluate whether the fitting is appropriate.
7. In Figure 3, the ANS profiles seem to comprise more than one state and are likely to be the sum of at least two states centered at around 1 and 2 M GdnHCl?
8. CD signals are recommended to report in molar ellipticities. The quality of the far-UV CD spectra is not enough. High concentration of denaturants may affect the signals at low wavelengths, generally below 210 nm. However, the low signal-to-noise seems to be affected by other factors. The authors can try to adjust the measuring parameters such as the slit width and number of repetitions. Furthermore, the authors mentioned that all chemicals were Sigma reagent grade. To my experience, GdnHCl with an ultrapure grade will produce much better signals that the lower grades. Note that the impurities or contaminated solvents will greatly affect spectroscopic measurements. Before spectroscopic measurements, the samples should be filtered and degassed. Detailed guidelines of spectroscopic measurements can be found in the paper by Kelly et al. ^2^
9. The units of the fluorescence intensities in Figures 2 and 3 are quite different. Are these data normalized by the same method? Furthermore, the spectra of N in panels E-2 should refer to the state in the absence of denaturant. Why the spectrum of N in Figure 2E-2 is different from that in Figure 3E-2? To me, the spectrum of N in Figure 2E-2 is strange. Please verify that the solutions are not affected by contaminants.

I confirm that I have read this submission and believe that I have an appropriate level of expertise to confirm that it is of an acceptable scientific standard, however I have significant reservations, as outlined above.

## References

[1] : Differences in the pathways of proteins unfolding induced by urea and guanidine hydrochloride: molten globule state and aggregates. *PLoS One*.2010;5(11) : 10.1371/journal.pone.0015035 e15035 10.1371/journal.pone.0015035 21152408PMC2994796

[2] : How to study proteins by circular dichroism. *Biochim Biophys Acta*.2005;1751(2) : 10.1016/j.bbapap.2005.06.005 119-39 10.1016/j.bbapap.2005.06.005 16027053

# Peer review: Reviewer response for version 1

**DOI:** 10.5256/f1000research.20788.r50821

**Published:** 2019-07-08

**Authors:** Vladimir Uversky

**Affiliations:** 1 Department of Molecular Medicine, College of Medicine, University of South Florida, Tampa, FL, USA

In this manuscript, Gurprit Sekhon and Ranvir Singh describe the results of the equilibrium unfolding of human aldose reductase. The authors conclude that urea- and GuHCL-induced unfolding of this protein is characterized by the presence of partially folded intermediate. This is an interesting study with some potential. However, additional experiments are needed to provide more evidence for the existence of an intermediate state and also to provide better description of the structural properties of this protein. 

1. The authors are encouraged to add near-UV CD spectroscopy to the arsenal of techniques utilized in their study. This will give very important information on the effect of denaturants on the tertiary structure of a protein. In fact, combined use of near- and far-UV spectroscopy is the accepted practice in studies on the conformational stability of proteins.
2. Quality of the reported far-UV CD spectra is very low. Spectra are very noisy and their utilization for the analysis of the effects of urea and GuHCl on secondary structure of human aldose reductase is questionable. The authors have to change settings to generate more reliable and less noisy spectra. Probably, protein concentrations should be increased too. 
3. Far-UV CD spectra of completely unfolded forms induced by high concentrations of urea and GuHCl are very different, suggesting that these unfolded forms are not similar. Why? 
4. Compactness of an intermediate state should be evaluated. This can be done by a whole host of hydrodynamic techniques.
5. The authors should make sure that the protein does not aggregate at the conditions promoting formation of a partially unfolded intermediate. This can be done by simple light scattering experiments. 
6. Data shown in Figure 3 suggest that very low GuHCl concentrations cause very noticeable changes in some of the analyzed parameters. Similar behavior was described previously ^1^ and was attributed to the aggregation of partially unfolded species. This phenomenon should be discussed.
7. The presence of partially folded intermediates can be visualized using "phase diagram" method described in previous work by Kuznetsova et al. ^2^ The authors are encouraged to use this approach for the analysis of their data.
8. The authors are encouraged to reconsider the use of terms "denatured/denaturation" and "unfolded/unfolding". Typically, unfolding is attributed to the process resulting in the formation of coil-like conformation, whereas denaturation is referred to the elimination of functional tertiary structure. Obviously, these terms are not equivalent - molten globule is denatured, but is not unfolded. Furthermore, although temperature increase typically causes melting of a protein tertiary structure, temperature-denatured species are often rather compact and preserve high levels of secondary structure. 
9. The manuscript contains some linguistic issues and errors and definitely needs editing.  

I confirm that I have read this submission and believe that I have an appropriate level of expertise to confirm that it is of an acceptable scientific standard, however I have significant reservations, as outlined above.

## References

[1] : Differences in the pathways of proteins unfolding induced by urea and guanidine hydrochloride: molten globule state and aggregates. *PLoS One*.2010;5(11) : 10.1371/journal.pone.0015035 e15035 10.1371/journal.pone.0015035 21152408PMC2994796

[2] : Use of the phase diagram method to analyze the protein unfolding-refolding reactions: fishing out the. *J Proteome Res*.3(3) :485-94 1525343010.1021/pr034094y

# Peer review: Reviewer response for version 1

**DOI:** 10.5256/f1000research.20788.r50315

**Published:** 2019-06-24

**Authors:** Jonathan W. Mueller

**Affiliations:** 1 Institute of Metabolism and Systems Research (IMSR), University of Birmingham, Birmingham, UK

Drs Sekhon and Singh have presented an in-vitro study "Human aldose reductase unfolds through an intermediate" to F1000Research to be considered for indexing. Foremost, the authors should describe the newly described intermediate more with respect to its potential physiological relevance to stress the relevance of this study.

 Please remove "ultra" from any description for protein crystal structures, but add the actual resolution of that structure, 0.66 A from Ref. 5, and explain what additional features were derived from these, compared to average crystal structures.

 "Understanding the capability of a polypeptide..." - this sentence only has limited linkage to the sentence beforehand and should be deleted. Instead, novel concepts of protein folding and stability should be briefly covered. ^1^^, ^^2^

 Figure 1:

- A) it is not clear what the blue and red dots mean.
- B) Please do not use green and red as colors.

Figure 2:

- A+B) Please label the different graphs within the diagrams.

The manuscript needs careful copy-editing.

I confirm that I have read this submission and believe that I have an appropriate level of expertise to confirm that it is of an acceptable scientific standard, however I have significant reservations, as outlined above.

## References

[1] : Generalized View of Protein Folding: In Medio Stat Virtus. *J Am Chem Soc*.2019;141(6) : 10.1021/jacs.8b10779 2194-2200 10.1021/jacs.8b10779 30566837

[2] : Melting Down Protein Stability: PAPS Synthase 2 in Patients and in a Cellular Environment. *Front Mol Biosci*.2019;6: 10.3389/fmolb.2019.00031 31 10.3389/fmolb.2019.00031 31131283PMC6509946
